# Biological Inspirations: Iron Complexes Mimicking the Catechol Dioxygenases

**DOI:** 10.3390/ma14123250

**Published:** 2021-06-12

**Authors:** Karolina Kałduńska, Anna Kozakiewicz, Magdalena Wujak, Andrzej Wojtczak

**Affiliations:** 1 Faculty of Chemistry, Nicolaus Copernicus University in Toruń, Gagarina 7, 87-100 Toruń, Poland; akoza@umk.pl; 2 Faculty of Pharmacy, Nicolaus Copernicus University in Toruń, Collegium Medicum in Bydgoszcz, Jurasza 2, 85-089 Bydgoszcz, Poland; mwujak@cm.umk.pl

**Keywords:** biomimetics, catechol dioxygenase, iron complexes

## Abstract

Within the broad group of Fe non-heme oxidases, our attention was focused on the catechol 1,2- and 2,3-dioxygenases, which catalyze the oxidative cleavage of aromatic rings. A large group of Fe complexes with N/O ligands, ranging from N_3_ to N_2_O_2_S, was developed to mimic the activity of these enzymes. The Fe complexes discussed in this work can mimic the intradiol/extradiol catechol dioxygenase reaction mechanism. Electronic effects of the substituents in the ligand affect the Lewis acidity of the Fe center, increasing the ability to activate dioxygen and enhancing the catalytic activity of the discussed biomimetic complexes. The ligand architecture, the geometric isomers of the complexes, and the substituent steric effects significantly affect the ability to bind the substrate in a monodentate and bidentate manner. The substrate binding mode determines the preferred mechanism and, consequently, the main conversion products. The preferred mechanism of action can also be affected by the solvents and their ability to form the stable complexes with the Fe center. The electrostatic interactions of micellar media, similar to SDS, also control the intradiol/extradiol mechanisms of the catechol conversion by discussed biomimetics.

## 1. Introduction

Compounds containing aromatic rings play an essential role in the natural carbon cycle. The biosynthesis and conversion of aromatic compounds are an integral part of this cycle. Nowadays, due to anthropogenic activities and industrial development, we are witnessing a massive increase in the number of aromatic ring compounds, including xenobiotic substances. Importantly, new compounds that reach the natural environment become more stable and challenging to convert or remediate. Over the last decades, compounds that mimic enzymes catalyzing the oxidative cleavage of aromatic rings (biomimetics) have been receiving more and more attention to apply them in biotransformation processes [1].

The degradation of aromatic compounds from both natural and human-made sources is mainly accomplished by microorganisms via aerobic (oxygen-requiring) or anaerobic (non-oxygen-based) pathways. The aerobic degradation includes oxidation to dihydroxyaromatic compound followed by oxidative cleavage of the aromatic ring. The anaerobic degradation involves the reductive hydrogenation of the aromatic ring resulting in the production of cyclohexane derivative and its subsequent fragmentation [2].

The present work provides a comprehensive literature review on biomimetic oxidation catalysts for aerobic conversion of catechol and activation of dioxygen, which are catalyzed by catechol dioxygenases. We focus on two metalloenzymes using non-heme iron ions as cofactors—catechol 1,2-dioxygenase (EC 1.13.11.1) and catechol 2,3-dioxygenase (EC 1.13.11.2), and analyze the catalytic activity of numerous Fe complexes mimicking the active center of these enzymes. Besides the ligands discussed in this article, the literature describes a wide range of ligands with the postulated catalytic activity initially confirmed by their characteristics and redox potential, but these reports are not in the scope of our paper. However, it should be mentioned that iron complexes, due to their redox properties, are used not only as biomimetics of catechol dioxygenases. In literature, there are known mononuclear [3] and multinuclear iron complexes capable of oxidizing cyclohexane [4,5] and oxidative conversion of alcohols to benzamides [6].

In our review, we will discuss the mechanisms of action of intradiol and extradiol catechol dioxygenases. Then, we will present the ligand structures divided into tri-, tetraand pentadentate. The review concerns iron complexes with N/O-donor ligands. However, Fe complexes with S-donor ligands are also reported [7].

## 2. Mechanism of Catechol Oxidative Cleavage

**Figure 1:**
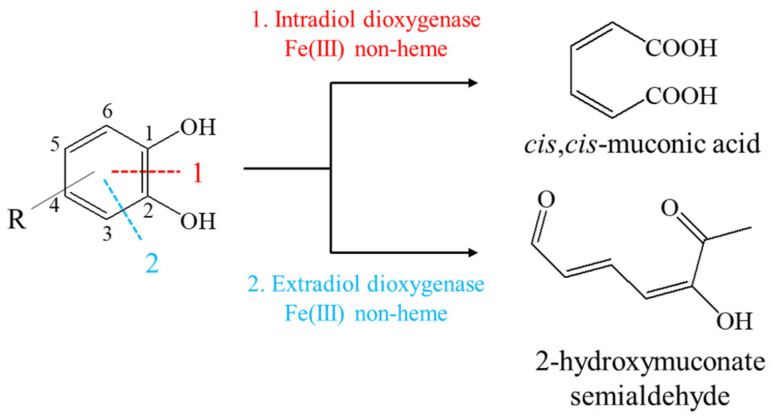
Structure of catechol where the C1–C2 bond cleavage site is marked red, and the decay of C2–C3 is marked blue.

Depending on the mechanism of the catalytic reaction, catechol dioxygenases are divided into intradiol and extradiol dioxygenases. Catechol 1,2-dioxygenase (1,2-CD) and protocatechuate 3,4-dioxygenase (3,4-CD) belong to intradiol dioxygenases, and catechol 2,3-dioxygenase (2,3-CD) to extradiol dioxygenases [8,9]. In our work, we focus on the presentation of 1,2-CD and 2,3-CD and their chemical models. Catechol and its derivatives possess two hydroxyl groups on adjacent carbon atoms in the aromatic ring. In the extradiol and intradiol catechol cleavage pathways, the different carbon-carbon bonds are cleaved by these enzymes. The 1,2-CD enzymes cleave the C1–C2 bond, and 2,3-CD cleave the C2–C3 bond in the aromatic ring (Figure 1) [10,11]. Dioxygenase cofactors differ in the oxidation state of the iron ion. In 1,2-CD, there is oxidized ferric iron depicted as Fe (III) (Fe^3+^), whereas in 2,3-CD, iron exists in a reduced ferrous state as Fe (II) (Fe^2+^) [12]. The iron complexes discussed in this work can mimic the intradiol/extradiol catechol dioxygenase reaction mechanism.

### 2.1. Intradiol Dioxygenase

Intradiol dioxygenases catalyze the aromatic ring cleavage at the ortho position to yield cis,cis-muconic acid (Figure 1). In this mechanism, the carbon–carbon bond between the catechol hydroxyl groups is cleaved, and subsequently, the hydroxyl groups are oxidized to the carboxyl groups [11,13].

**Figure 2:**
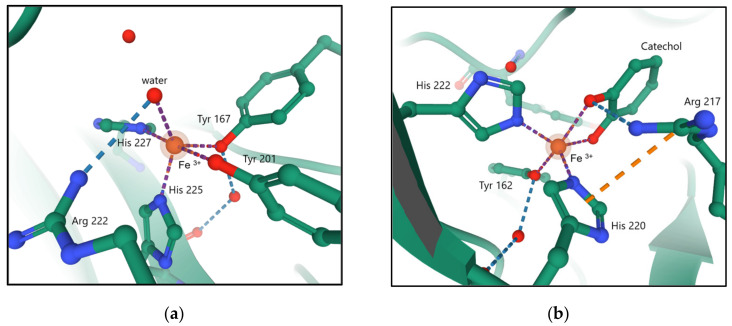
The active center of 1,2-CD with a coordinated water molecule (**a**) [PDB–2XSR] [14] as compared to the active 1,2-CD site that coordinates the catechol molecule (**b**) [PDB–3HHY] [15].

Catechol 1,2-dioxygenase most often function as homodimers. Figure 2 shows the active center of 1,2-CD with coordinated water and bound catechol as substrate.

**Figure 3:**
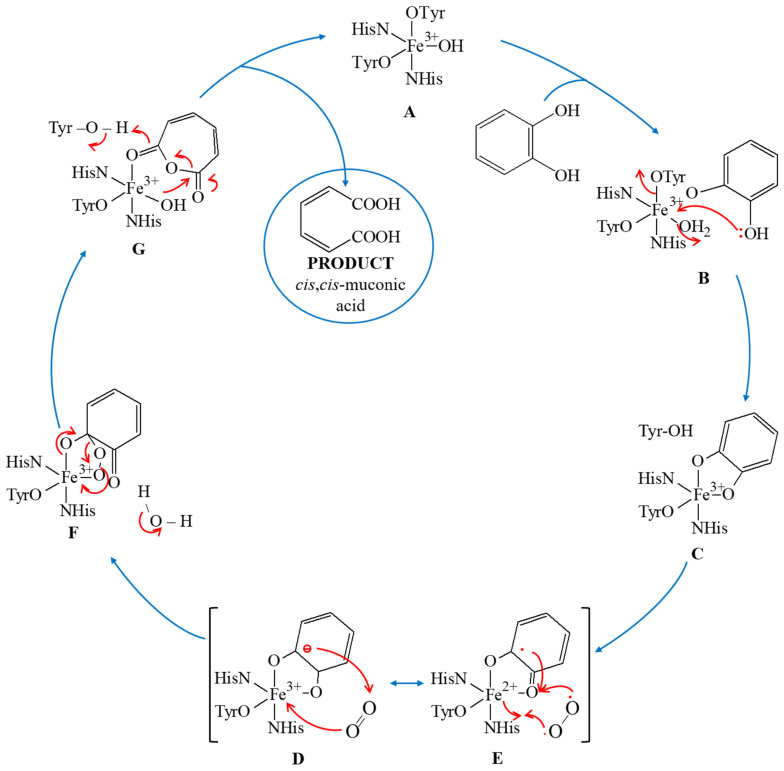
The overall mechanism of intradiol catechol deoxygenation catalyzed by 1,2-CD.

The cofactor of intradiol dioxygenase (Fe^3+^) is stabilized by two Fe–O_Tyr_ bonds and two Fe–N_His_ bonds. The fifth metal-linked ligand is the hydroxyl group derived from water deprotonation [15,16]. In 1,2-CD, iron (III) has a tetragonal pyramid-shaped coordination sphere. Tyrosine Tyr-201 and histidine His-227 bind axially, and Tyr-167 binds equatorially to form a plane. The fourth ligand is His-225, which stabilizes iron (III) by binding equatorially. The numbering of the individual residues corresponds to that used in the structure of the *Acinetobacter radioresistens* protein [14]. Transition metal centers in different oxidation states may exhibit distinct mechanisms in substrate oxidation. The overall catechol dioxygenase reaction mechanism is shown in Figure 3. In the presence of catechol or its derivatives, both the axially bound tyrosine residue and equatorially bound hydroxyl group leave the coordination sphere (A). The substrate present in anionic form can bind bidentately into both vacant axial and equatorial positions (B). This coordinated iron has a free space in a square-pyramidal coordination sphere that can act as a pocket for binding and activation of molecular oxygen (C). The molecular oxygen is activated by an electron from the bound substrate and transformed into a more reactive form, the superoxide radical (O_2_^−•^), which can form ferric-peroxo species with Fe (III) (D, E). The substrate present in the enzyme′s active center coordinates to Fe (III) via a peroxide bridge. Substrate binding to the Fe (III) coordination sphere shows a remarkable ability to change the manner of coordination with metal.

As a result of intramolecular electron transfer, one of the hydroxyl groups is converted into a ketone group. This conversion allows the previously dissociated Tyr residue to bind again with the iron (III) cofactor in an anionic manner. Dissociated H^+^ can combine with one of the oxides on the peroxide bridge, thus stabilizing the Fe (III) (F). In the next step, the bond between the bridging oxygenates is broken, and the hydroxyl group and the oxidized muconic anhydride substrate are not connected (G). The final stage of the catalytic mechanism involves the release of the product and regeneration of the active site to its initial state (A) [16].

### 2.2. Extradiol Dioxygenase

In the extradiol catechol dioxygenase reaction mechanism, the bond C2–C3 in the aromatic ring is cleaved (Figure 1). Catechol is converted by 2,3-CD to 2-hydroxymuconate semialdehyde. One of the hydroxyl groups on the second carbon (C2) is oxidized to the carboxyl group, while the group on the third carbon (C3) is converted to the carbonyl group [9].

**Figure 4:**
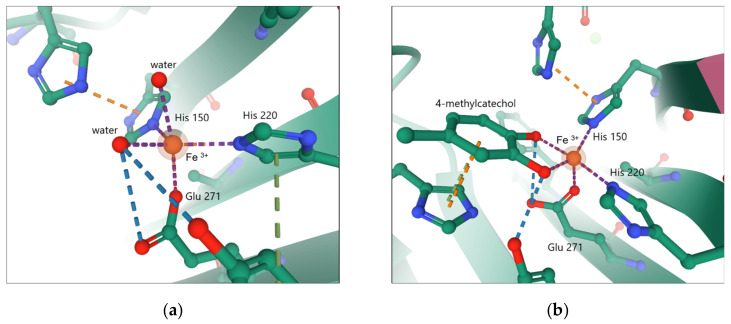
The active center of 2,3-CD with two coordinated water molecules [PDB code 5ZSZ] (**a**) as compared to the active 2,3-CD site that coordinates the 4-methycatechol molecule [PDB code 5ZNH] (**b**).

In the active site of 2,3-CD the coordinated Fe (II) iron builds five- or six-coordination complexes, with two histidine residues bound in the equatorial plane and glutamic acid bound axially. Water molecules occupy the remaining positions. Figure 4a shows the trigonal bipyramidal coordination sphere of cofactor, while Figure 4b presents the active center of 2,3-CD with bound substrate molecule.

**Figure 5:**
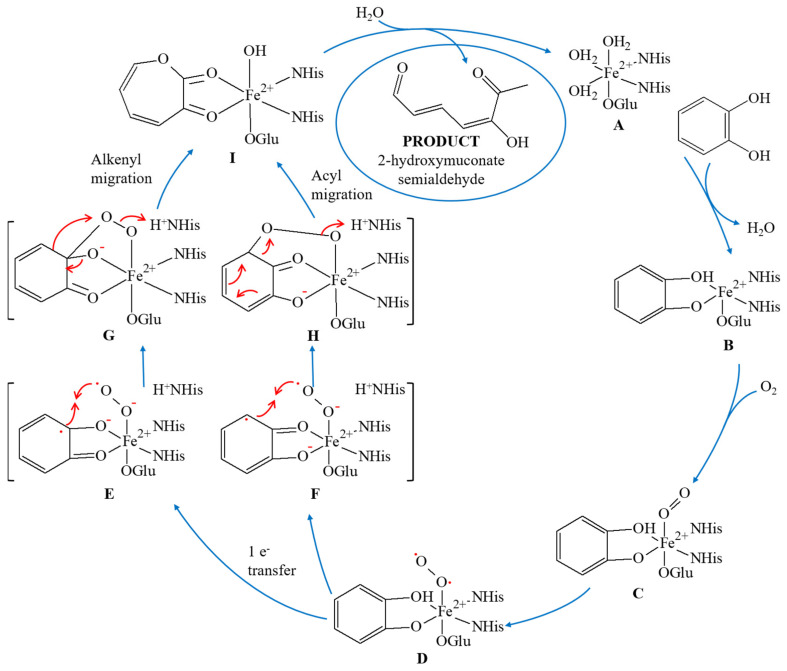
Mechanism of catechol conversion by 2,3-CD extradiol cleavage.

Figure 5 presents the mechanism of extradiol catechol dioxygenase conversion. In the resting state of the 2,3-CD enzyme, cofactor Fe (II) is stabilized by two Fe–N_His_ bonds, one Fe–O_Glu_ bond, and two or three bonds with water molecules (A). In the presence of a substrate, all coordinated water molecules are replaced by a bidentate catechol monoanion coordinated with the Fe (II) center at the equatorial position (B). The oxygen molecule is attached to Fe (II) in a free axial position and is activated to superoxo species (C, D). 

Then, the intramolecular electron transfer from Fe (II) to the O_2_ molecule causes the cofactor oxidation to Fe (III). The Fe (III) reduction to ferrous iron occurs with the electron from the catechol anion, with the formation of semiquinone-Fe (II) superoxide intermediates (E, F). In the next step, superoxide intermediates are reduced to the corresponding bridging peroxide forms (G, H). Within the peroxide forms, a migration of alkenyl or acyl occurs (Criegee rearrangement), leading to lactone as a standard product (I). Finally, due to lactone hydrolysis mediated by a metal-coordinated hydroxide group, 2-hydroxymuconate semialdehyde is produced and released from the enzyme active site [2].

## 3. Iron Complexes Mimicking Catechol Dioxygenases

Biomimetic complexes generally have the first coordination sphere similar to the enzyme they mimic. Concerning catechol dioxygenases, iron complexes should have spheres consisting of N and O donor ligands [17]. Moreover, apart from iron, which occurs naturally in 2,3-CD and 1,2-CD enzymes, complexes of other d-electron metals, such as Cu, V, Mn, and Ni, have been designed, but are not discussed in this article [18,19,20,21,22,23].

The first data reported in the literature involved the chemical characterization and evaluation of the biomimetic activity of Fe complexes. These reports have shown a strong correlation between the iron oxidation state and the proposed mechanism. However, a growing body of literature has recently demonstrated that the reaction mechanism can be controlled by changing the reaction conditions [24,25,26].

Studies on the catalytic activity of the iron complexes were mainly carried out with the use of 3,5-di-*tert*-butylcatechol (3,5-DTBC) as a test substrate. It should be emphasized that one of the initially reported dependencies between the activity of the complexes and their structure is the increase in reactivity towards dioxygen correlated with the rise in Lewis acidity of the Fe (III) center [27]. Some studies have been extended to investigate the effect of different substituents on the catechol conversion efficiency and product specificity.

The reaction course was examined, among others, by monitoring the ligand-to-metal charge transfer (LMCT) bands characteristic for the studied complex. It was shown that for Fe (III)—catechol adducts, two LMCT bands are related to the charge transfer from the catecholate anion to the central atom. The observed red-shifted LMCT bands were associated with increased catalytic activity [12]. The steric hindrance around the cofactor correlates with a decrease in the reaction rate. Hitomi and coauthors experimentally confirmed that the energy for the lower energy LMCT band correlates with the reaction rate constant for catechol oxidation [12].

**Figure 6:**
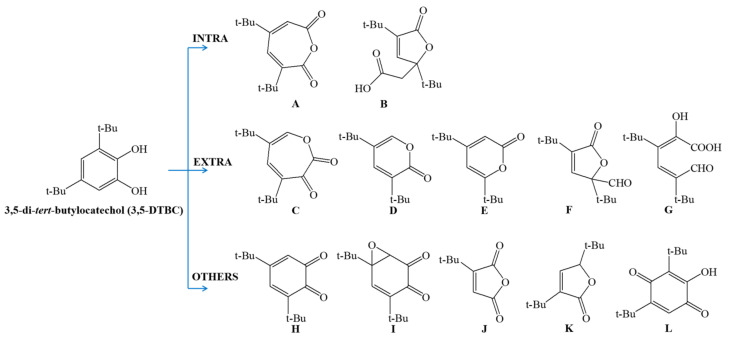
Products of catechol oxidation in 3,5-DTBC: 3,5-di-*tert*-butyl-1-oxacyclohepta-3,5-diene-2,7-dione (**A**), 3,5-di-*tert*-butyl-5-(carboxymethyl)-2-furanone (**B**), 4,6-di-*tert*-butyl-1-oxacyclohepta-4,6-diene-2,3-dione (**C**), 3,5-di-*tert*-butyl-2H-pyran-2-one (**D**), 4,6-di-*tert*-butyl-2H-pyran-2-one (**E**), 3,5-di-*tert*-butyl-5-(formyl)-2-furanone (**F**), cis,cis-3,5-di-*tert*-butyl-2-hydroxy-muconic semialdehyde (**G**), 3,5-di-*tert*-butyl-1,2-benzoquinone (**H**), 3,4-epoxy-3,4-dihydro-4,6-di-*tert*-butyl-1,2-benzoquinone (**I**) 3-*tert*-butylfuran-2,5-dione (**J**), 3,5-di-*tert*-butylfuran-2(5H)-one (**K**), 3,5-di-*tert*-butyl-2-hydroxy-1,4-benzoquinone (**L**).

Analysis of data on catalytic activity showed that biomimetics convert 3,5-DTBC according to both extradiol and intradiol catechol cleavage mechanisms, but their classification is based solely on the main cleavage products identified after the reaction. Based on the literature reports, the most frequently reported products of the catechol conversion are summarized in Figure 6.

In the intradiol cleavage mechanism, the substrate is bound via one phenolate oxygen atom before a dioxygen attack. The free pocket remaining in the iron coordination sphere is ready to adopt an oxygen molecule. By contrast, in the extradiol cleavage mechanism, the substrate is coordinated via both phenolic oxygen atoms. From the beginning, there is a free pocket in the coordination sphere ready for dioxygen binding. The use of tetradentate ligands will force the formation of “intradiol” products. For tridentate ligands, a mixture of products is usually obtained. The formation of a particular product depends on the mode of ligand coordination with the metal center and oxidation conditions. For *fac* isomers, extradiol cleavage is more preferred. For *mer* isomers, the primary mechanism is intradiol, but these biomimetic complexes can also oxidize the substrate according to other mechanisms, e.g., auto-oxidation [28].

### 3.1. Small Molecular Complexes Mimicking Catechol Dioxygenases

To the best of our knowledge, studies published in 1979 by Funabiki et al. were the first reports on compounds mimicking the catechol dioxygenase activity [29]. The authors carried out the experiment in which they used iron (II) chloride solution with 2,2′-bipyridine and pyridine as nitrogen atom donors under atmospheric oxygen to the conversion of catechol. [29]. In further studies, the authors demonstrated the formation of different products via all mechanisms (Figure 6). The main cleavage product (H) was formed via the other mechanism, and the amount of the intradiol mechanism products (A and B) was larger than those of the extradiol mechanism (F) [30,31]. Studies by Funabiki et al., published almost 20 years later, provided deeper insight into the previous findings by describing the iron (III) binuclear complex structure with catecholate and chlorides in the coordination sphere. They confirmed that the obtained complex (without N donor atoms) is too stable to react with O_2_. In the presence of a small amount of pyridine as the N-atoms donor, chloride anions were exchanged for pyridine ligands, resulting in the complex activation [32]. In other studies on complexes with biomimetic properties, the same research group confirmed that the presence of the LMCT band correlated with the nature of Fe-catechol complexes [30,33,34].

Biomimetic compounds, able to convert catechol via the 2,3-CD and 1,2-CD mechanisms, can be divided according to the way of ligand coordination: (1) tetradentate: linear, planar, tripodal, macrocyclic ligands; (2) tridentate: meridional and facial ligands; (3) other ligands such as pentadentate ligands.

#### 3.1.1. Tetradentate Ligands

**Figure 7:**
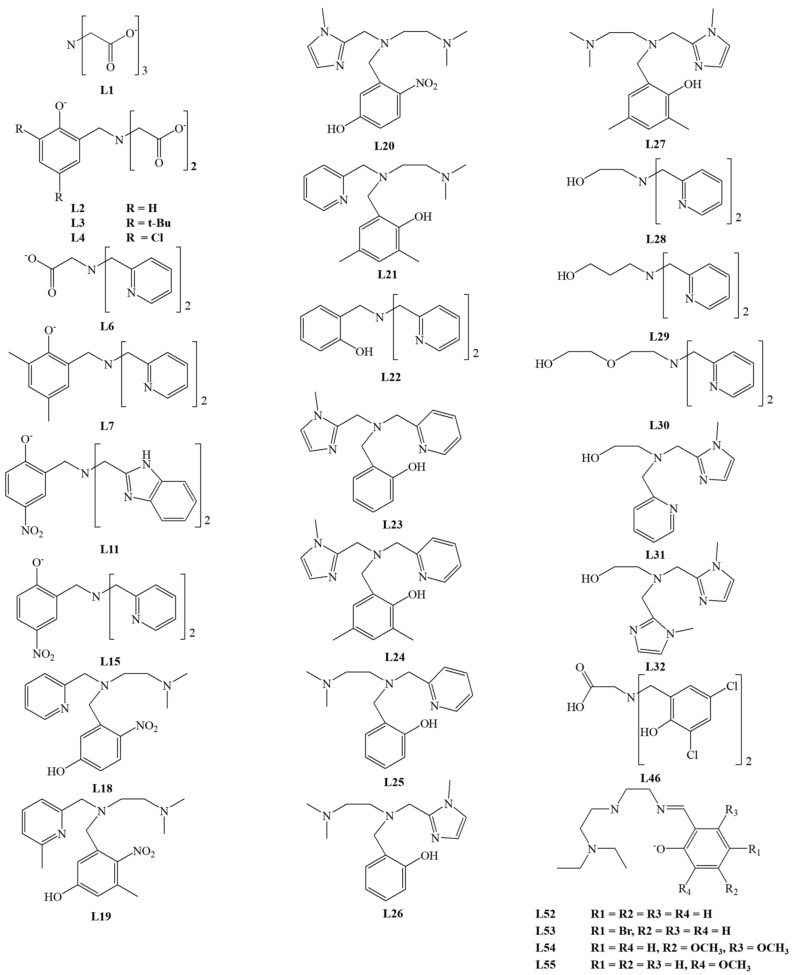
Tetradentate **N_3_O** and **NO_3_** donor ligands.

**Figure 8:**
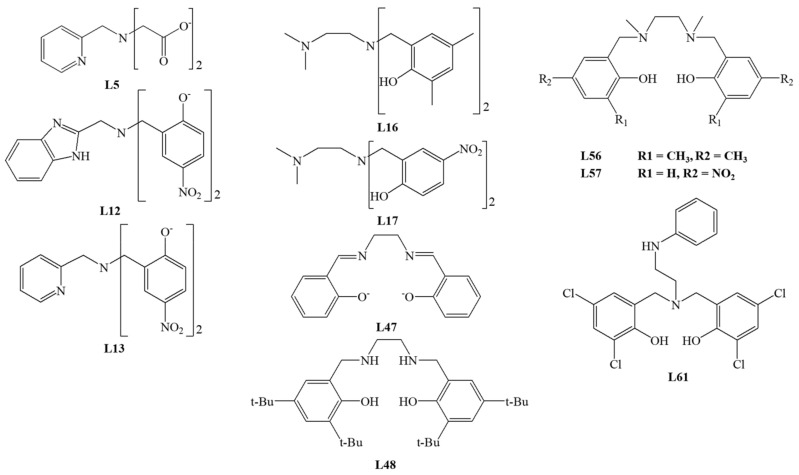
Tetradentate **N_2_O_2_** donor ligands.

**Figure 9:**
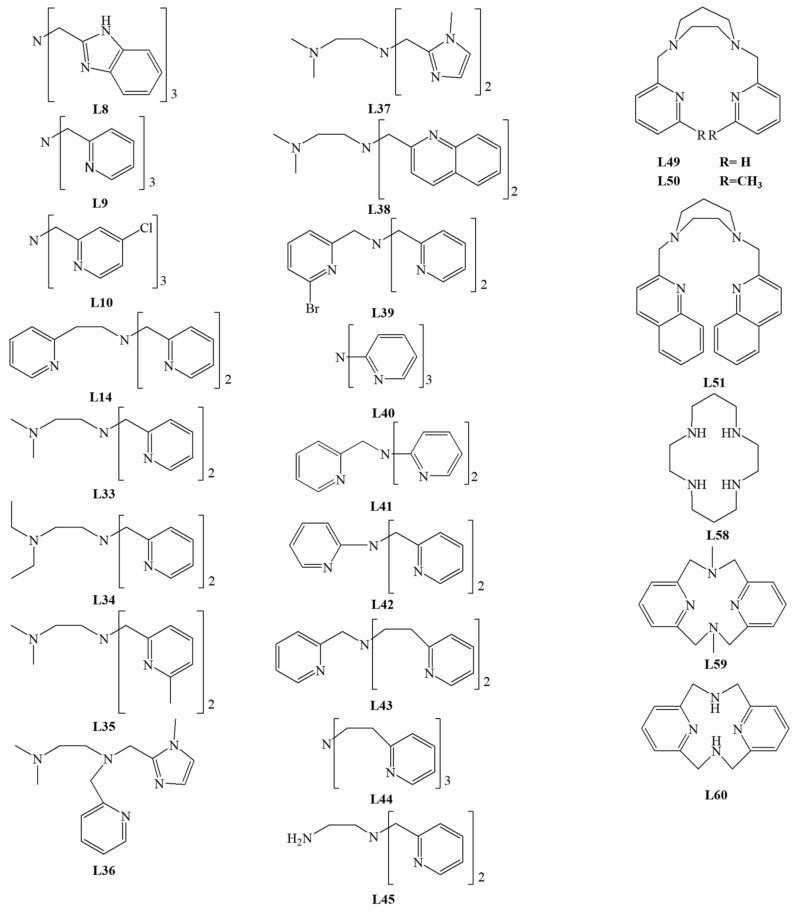
Tetradentate **N_4_** donor ligands.

The largest group of studied ligands are tetradentate N/O donor ligands. Among the tetradentate ligands, the **N_4_**, **NO_3_**, **N_2_O_2_**, **N_3_O** donor ligands were investigated. The structure of ligands is presented in Figure 7, Figure 8 and Figure 9.

Iron complexes with tetradentate tripodal ligands are a large group of compounds demonstrating biomimetic activities. Their structure allows them to coordinate well to a metal center and to create a free pocket to bind the catecholate ion. The scientific interest in these ligands as potential models of catechol dioxygenase started in the 1980s. The first complex was described in 1982 by Weller et al., who demonstrated a catalytic activity of the iron (III) complex with the **L1** ligand. The ring cleavage reaction was carried out in the presence of dioxygen in aqueous borate buffer pH 8.5, mixed with DMF (dimethylformamide) or CH_3_OH. Within 4 to 7 days, they obtained approximately 80% of the 3,5-DTBC conversion to intradiol cleavage products **A** and **B** (Figure 6) [35]. Two years later, White et al. published structural studies on the iron (III) complex with the same ligand. Based on structural studies, they proposed a mechanism of catecholate aerobic oxidation, where iron (III) is involved in the whole process of oxidation, not only in the dioxygen activation [36].

Que and colleagues reported an interesting group of ligands derived from **L1**. In their studies, they replaced one (**L2**–**L5**), two (**L6**), or three (**L7**–**L9**) acetic acid residues in **L1** with N donor ligands [27,37,38,39]. For all these compounds, intradiol cleavage products have been identified. The obtained results indicated that the yield of the process depends on the strength of the Lewis base (ligand), which affects the acidity of the metal–iron (III). As the Lewis acidity of the metal increases, the coordination bond strength of catecholate increases. With over 90% of the intradiol products, the best results were obtained for complexes with **L9** (in CH_3_CN), **L7,** and **L5** ligands in DMF.

X-ray analysis confirmed the architecture of iron (III) complexes with ligands **L6** [27] and **L9 [38]**. The tetradentate tripodal ligand is bound in both complexes, leaving two empty positions: equatorial and axial, where 3,5-DTBC can bind [27,38]. Hitomi et al. studied the iron (III) complexes with **L9** ligand and various catecholate anions. They showed a linear correlation between the bathochromic shift of the low-energy LMCT band and the increasing logarithm of the oxidation rate constant [12].

Jastrzębski et al. compared the experimental results and the DFT calculations for the iron (III) complexes with **L9** and its halogen derivative **L10**. Their research confirmed the significant influence of electron effects from catechols and ligands on the conversion mechanism. The authors proposed a mechanism related to monodentate binding of substrate and a vacancy formation in the coordination sphere for molecular oxygen binding. They found that the rate-limiting stage is the formation of the carbon–oxygen bond [40]. Moreover, Jastrzębski et al. reported the use of iron (III) complex with the **L9** ligand to produce dimethyl adipate with efficiency of 60%. They found an increase in the reaction rate associated with an increase in temperature in the range 30–80 °C [41].

Viswanathan et al. confirmed the relation between the Lewis acidity of the metal and catechol conversion capacity in the research on tripodal ligands containing pyridine derivatives and o-nitrophenol (**L8,L9,L11**–**L15**). The reaction was carried out according to standard procedures in CH_3_NO_2_ as a solvent, mixing a substantial excess of 3,5-DTBC with corresponding iron (III) complexes. Product analysis indicated an intradiol cleavage mechanism, and complexes with **L11**–**L13** ligands gave the best results [42]. The amount of intradiol product obtained for the complex with the **L9** ligand was half of that reported by Jang and colleagues [38]. The crystals were obtained for the Fe (III) complex with **L15**, and the molecular structure was confirmed using X-ray analysis [42].

In 2003, Velusamy and colleagues described other groups of compounds with **L16** and **L17** ligands. The catalytic activity studies were carried out in DMF solution with the addition of piperidine, use of atmospheric oxygen, and 3,5-DTBC as the model substrate. For the **L17** complex, 86% of intradiol products were obtained, and 14% of **E** product were identified (Figure 6). This Fe complex has a five-coordinated center, which is similar to 1,2-CD [43]. Subsequent modifications allowed to obtain several ligands of which only **L18**, **L19**, **L20**, and **L17** showed catalytic activity [44,45], with the best results achieved for the iron (III) complex with **L18** (about 75–80% intradiol products). The X-ray structure analysis of Fe (III) complexes indicated that properly selected hindered bisphenolate ligands can stabilize the metal center. As a result, the complex may mimic the catalytic center of 1,2-CD or 3,4-CD. It is consistent with results obtained for complexes with **L16** and **L18** [43].

Mayilmurugan et al. described the iron (III) complex with the new **L21** ligand. They found that the presence of substituents affecting the Lewis acidity of the coordination center determined the appearance of extradiol products. For the complex with **L21**, the influence of reaction conditions on the 3,5-DTBC cleavage was investigated. Interestingly, changing the solvent (CH_3_OH, CH_3_CN, DMF) and deprotonating base (Et_3_N, piperidine), and the removal of chloride ions (AgNO_3_) altered not only the cleavage mechanism towards intradiol but also the type of products formed. The presence of sterically demanding substituents in the ligand influenced the monodentate binding of catecholate, and the use of AgNO_3_ or a stronger base (piperidine) changed the coordination mode to a bidentate one. The architecture of the complexes also influenced the effectiveness of the reaction and the specificity of the product [46].

Another study by Mayilmurugan et al. reported a group of compounds containing the tetradentate monophenolate ligands (**N_3_O**). Ligands **L22**–**L27** contained phenol, pyridine, trimethylamine, and N-methylimidazole moieties. The cleavage reaction was performed for 3,5-DTBC in CH_3_OH. The X-ray analysis confirmed architecture iron (III) complexes with **L24** and with **L27**. The authors demonstrated that changing the Lewis base to a stronger one (pyridine to N-methylimidazole) leads to a decrease in iron (III) acidity, lowering the intradiol type conversion and an increase in reactivity towards extradiol cleavage. Research revealed that the presence of the N-methylimidazole moiety reduces steric hindrance and enhances the substrate binding to iron (III), and the introduction of a trimethylamine derivative increases the covalency of the Fe–catecholate bond, therefore affecting Fe (III) Lewis acidity [47].

Sundaravel et al. modified tripodal ligands containing pyridine, N-methylimidazole, and the -CH_2_R moiety, where R was the OH group or its derivatives (**L28**–**L32**). The authors investigated the catalytic activity of the obtained biomimetics in methanol solution with 3,5-DTBC as substrate and various bases (piperidine equivalents). For complexes with **L28**-**L32** ligands, a high percentage of intradiol products and a small yield of extradiol products were obtained. This preference is consistent with the intradiol mechanism expected for the six-coordination architecture of the complex with facially coordinated **N_3_** ligands. However, for complexes containing **L31** and **L32**, the percentage of intradiol products decreased for all piperidine equivalents compared to the complexes with **L28**-**L32**. The increase in the piperidine amount resulted in the increased rate of quinone products [48].

For the **L28** ligand, Li et al. obtained a binuclear complex with iron (III) efficiently converting 3,5-DTBC according to the intradiol mechanism in a methanol solution, with a small addition of piperidine, since the complex itself contains an ethoxy moiety—an internal base. This moiety, able to accept protons, efficiently mimics the tyrosine residue present in the dioxygenase catalytic center [49].

The promising results of studies on trimethylamine, pyridine, and N-methyimidazole derivatives have opened a further perspective for the synthesis of new biomimetic complexes, including those with a new group of **N_4_**-donor ligands **L33**–**L38**. For the Fe (III) complex with **L33**, the catalytic conversion of 3,5-DTBC was investigated in the acetonitrile solution with the trimethylamine (Et_3_N) addition. The analysis of the reaction mixture shows the formation of products via both intradiol (24–73% products) and extradiol (8–55% products) cleavage mechanisms. The presence of a strong base (N-methylimidazole) reduces the acidity of the metal center and favors the intradiol cleavage. The influence of the amount of Et_3_N on the ratio of intradiol to extradiol products was also investigated. A critical step determining the formation of extradiol products is the dissociation of the weakly coordinated group—NMe_2_, with the appearance of a vacant site in the semiquinone intermediate product. The ratio of cleavage products derived from these two different cleavage reactions depends on the properties and nature of the groups used in the tripodal ligand [50].

Merkel et al. modified one of the pyridine rings in tripodal TPA (Tris-(2-pyridylmethyl)amine), substituting bromine in the ortho position (**L39**). For new iron (III) complexes, the architecture of compounds was confirmed by X-ray structural studies. The substituted pyridine ring (internal base) was not bound directly to the iron (III) center in any of these complexes. The activity of these complexes was determined for 3,5-DTBC as a substrate, in acetonitrile as a solvent, and with the addition of piperidine. Results demonstrated that the products are derived according to the intradiol cleavage reactions. Such spatial arrangement suggests a good mimicry of the 1,2-CD cofactor and a tyrosine residue detaching in the presence of the substrate [51].

Merkel et al. studied the activity of **L9** derived tripodal ligands formed by shortening or extending the **L9** ligand arms. Ligands **L40**, **L41**, **L42** were formed by shortening the arm′s length by one carbon in one, two, and three arms, respectively, whereas ligands **L14**, **L43**, **L44** were obtained by extending the arm by one carbon, sequentially. The kinetic studies carried out in methanol solution with the addition of piperidine showed that any modifications of the chelate rings size decreased the activity of the biomimetic complex in comparison with **L9** [52]. Testing the catalytic activity of the iron (III) complex with **L45** under similar conditions revealed that it is less active than complex with **L9** [53].

Due to the redox properties, Fe (III) complex containing the tripodal ligand **L46**—a glycine derivative, has high efficiency in converting catecholate. The process was carried out with O_2_ access in various solvents (DMF, methanol, ethanol, acetonitrile) in the presence of triethylamine. The reaction efficiency was 85–98% [54].

Que et al. and Lauffer et al. demonstrated the catecholate conversion by iron complexes with disalicylalethylenediamine (salen) **L47** in reaction with O_2_ (in DMF and THF solution). The d-electron metal complexes of salen had attracted great interest due to their ability to bind molecular oxygen. The biomimetic activity in the catechol conversion was confirmed with furanone as the main product [37,55]. The reported results differed from those published by Que et al., where the main product amounted to 35% and resulted from the intradiol cleavage reaction [37].

Safaei et al. obtained and characterized the iron (III) complex with the **L48** ligand. The compound showed an excellent ability to convert 3,5-DTBC (100% conversion). The product analysis showed that the complex is more selective towards the extradiol cleavage mechanism (74%) [56].

Studies on iron (III) complexes with tetradentate ligands **L49**–**L51** showed their high efficiency against 3,5-DTBC as substrate [57]. In addition, the effect of solvent and base was examined. For the complex in which catecholate was coordinated via one oxygen atom, the highest conversion to extradiol products from 34.6% to 85.5% (Figure 6D,E) were observed for complexes with **L49**–**L51**. The complex of **L50** revealed an excellent selectivity of extradiol over intradiol pathway with E/I = 181 compared to 57 and 9 for complexes with **L49** and **L51**, respectively. For bidentate catecholates, the switch of the base from triethylamine to piperidine changed the reaction type towards the intradiol cleavage. Doubling the concentration of triethylamine resulted in the amount of intradiol products several times higher than extradiol products [57].

Research of tetradentate ligands based on salicylamide derivatives **L52**–**L55** by Wang et al. revealed that they act according to the intradiol mechanism. The cleavage reaction rate was highest for the **L53** complex and the lowest with **L54**, which is consistent with the increase in the Lewis acidity of the iron (III) center. The complex with **L53** showed the highest efficiency of about 70–80% for the substrate conversion to intradiol products. These results are in line with other reports and confirm the influence of the substituents in the ligand rings on the reactivity of the complex, which is related not only to electrochemical but also to steric properties of the ligand [58].

Velusamy et al. presented iron (III) complexes with tetradentate linear ligands **L56** and **L57**, which are derivatives of **L48**. For the iron (III) complex with **L57**, the reaction rate and selectivity were very high, with the formation of intradiol product A (Figure 6) exclusively. It is surprising to compare the reaction rates of the iron (III) complexes of **L57** with that of the tripodal **L17**, which is much higher for the linear ligand complex. That effect is interesting because the introduction of **L57** into the complex reduces the Lewis acidity of the coordination center; the opposite effect might be expected, with the reaction rate lower than for the Fe (III) complex of **L17** [43].

It is important to emphasize that the proper architecture of the first coordination sphere and desired electrochemical properties of the complex do not ensure the catalytic activity of the compound. Compounds with macrocyclic ligands, for example, **L58**, do not show catalytic activities despite good electrochemical properties. **L58** is a four-coordinated macrocyclic ligand, and upon its coordination, the pocket is too small to bind both substrates [59]. Interestingly, Koch et al. confirmed that macrocyclic ligands could form catalytically active iron (III) complexes [60]. They investigated the activity of the Fe (III) complex with **L59** in DMF and obtained a high conversion rate and 70% of intradiol product A (Figure 6). The contradictory results from studies on **L58** and **L59** complexes show that an essential intermediate step is the oxygen binding with the coordinated substrate [59,60].

The iron (III) complex with **L60,** similar to **L59,** reported by Raffard et al. showed a rapid conversion. For the first time, a complex with tetradentate macrocyclic ligand **L60** could cleave catechol according to both extradiol and intradiol mechanisms [61]. The ligands **L59** and **L60** differ from each other by the presence of methyl group or hydrogen on the bridging nitrogen, respectively. Stepanović et al. carried out DFT (Density Functional Theory) calculations, which indicate that the conversion follows both mechanisms in exothermic processes, but the intradiol process is thermodynamically favorable. The studies have demonstrated that both reaction mechanisms involve several spin intermediate states. The crucial element in the structure of both ligands seems to be the presence of a hydrogen atom capable of forming H-bonds, which results in a good stabilization of dioxygen [62].

The iron (III) complexes of tripodal tetradentate ligands catalyze the catechol conversion via the intradiol cleavage mechanism. However, Poureskandari et al. showed that the iron (III) complex with **L61** converts catecholate into extradiol products with an extremely high conversion efficiency of 99.6%. The reaction was carried out with the addition of triethylamine in methanol solution and 3,5-DTBC as substrate. More than 50% of the obtained products resulted from the C2–C3 bond cleavage and only 16% from the cleavage of the C1–C2 bond. The main conversion product was E (35.2%, Figure 6). It may be concluded that the ring substitution with chlorine atoms might be a key element determining the biomimetic properties and catalysis mechanism and reproducing the enzymatic activity of catechol dioxygenases [63].

High selectivity towards the intradiol mechanism is an undeniable advantage for tetradentate biomimetics. The vast majority of ligands have their tripodal architecture based on aromatic rings containing nitrogen atoms (pyridine and imidazole), which influence the Lewis acidity of the central atom. However, in case of N_4_ macrocyclic ligands, the extradiol mechanism is also possible, especially for ligands with relatively lower Lewis basicity of donor atoms. For mixed N/O ligands, the intradiol mechanism dominates in most cases, although the extradiol products could be obtained for tripodal ligands with significant steric hindrance related to the pendant. In addition, the effect of solvent could be used to control the regioselectivity of the catalyst.

#### 3.1.2. Tridentate Ligands

A suitable pocket for binding catechol anions, in addition to triploid ligands, is provided by facially and meridionally bonded ligands. Iron (III) complexes with facially bound ligands preferentially follow the extradiol cleavage reaction mechanism. In contrast, complexes with meridionally bound ligands convert the substrates predominantly through intradiol cleavage and unfavorable auto-oxidation mechanism.

**Figure 10:**
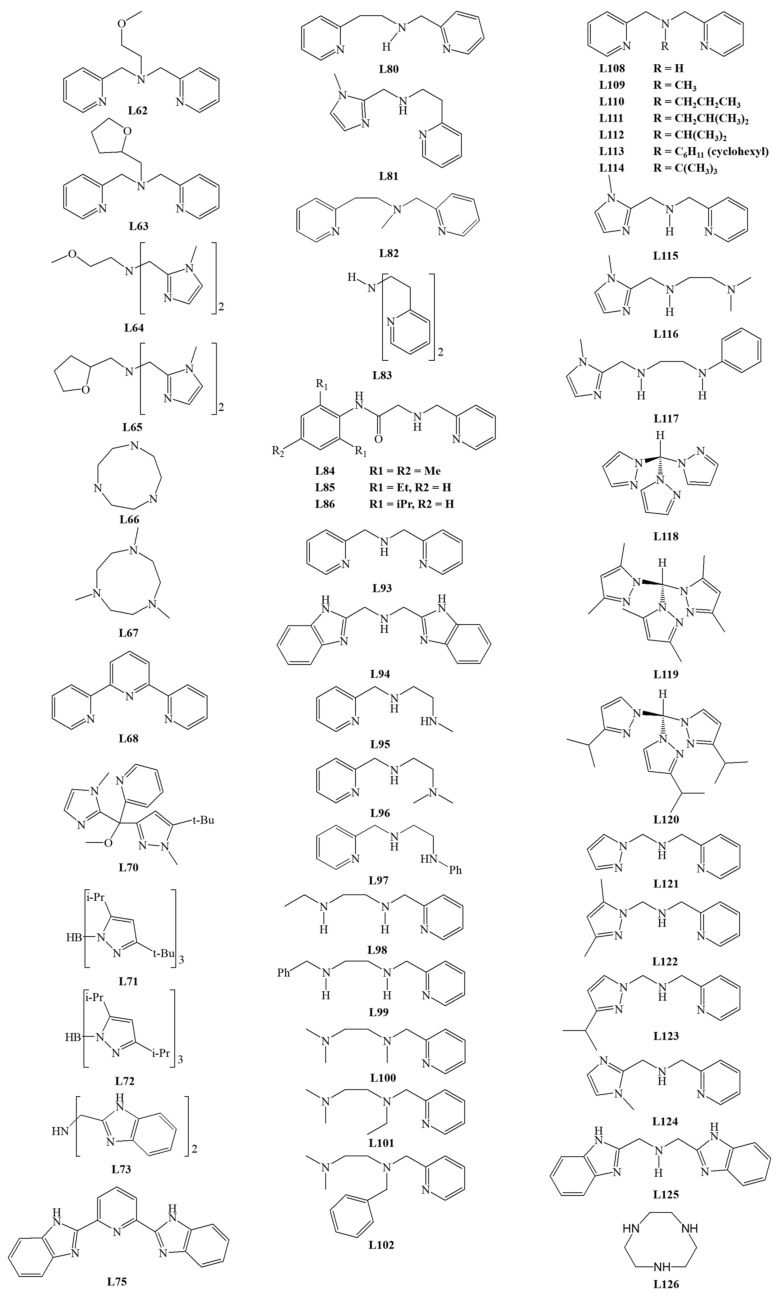
Tridentate **N_3_** ligands.

**Figure 11:**
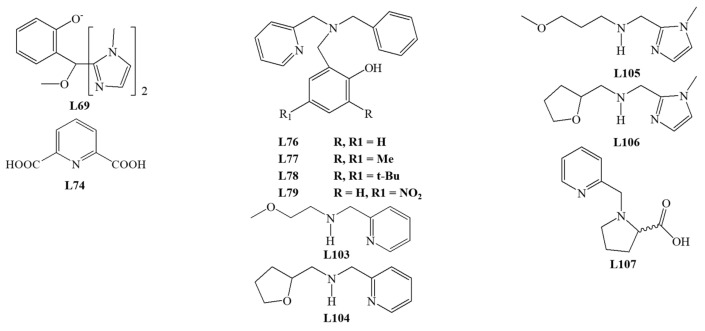
Tridentate **N** and **O** donor ligands.

**Figure 12:**
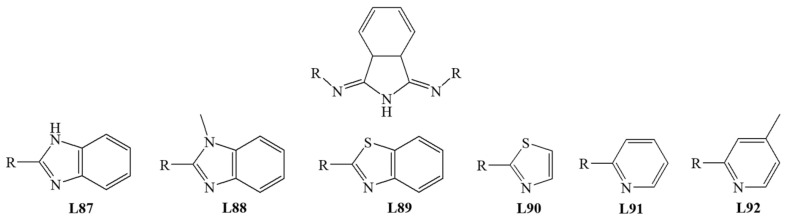
**N_3_** ligands—derivatives of isoindoline.

Among the tridentate ligands, the **N_3_**, **N_2_O**, and **NO_2_** type ligands have been investigated. The structure of ligands is presented in Figure 10, Figure 11 and Figure 12.

The iron (III) complex with **L21** is not the only one with a tripodal ligand capable of conversion by the extradiol mechanism. Sundaravel et al. showed that the Fe (III) complexes with the **L62**–**L65** ligands could convert catechol according to both extradiol and intradiol mechanisms. The highest ratio of the extradiol to intradiol products (E/I) was obtained for the complex with **L25** (E/I = 2.3). Complexes with **L62** or **L65** gave a higher percentage of the extradiol products, while for the complex with **L63**, more intradiol products were formed. The obtained results indicate that the oxygen of the ether group significantly accelerates the reaction [64].

Dei et al. also characterized the complex with the facial coordinated tridentate cyclic ligand **L66**, which is catalytically active. The activity studies performed in DMF, dichloromethane, and methanol indicated the formation of extradiol cleavage products with an efficiency from 35 to 70%. The intradiol products were detected only under conditions with methanol as a solvent, whereas for DMF solution, the yield of benzoquinone derivative (Figure 6H) was higher than extradiol products. These findings provide evidence that the properties (polarity) of the solvent significantly influence the mechanism of cleavage reaction [59]. Two other research groups tested the reactivity of the complex with **L66** under different conditions and with substituted catecholate substrates. The obtained results indicate the extradiol mechanism of the iron complex with **L66**, as well as the influence of solvent polarity and deprotonating base (derivative of piperidine or imidazole) [65,66].

Jo et al. studied the activity of iron (III) *fac* and *mer* complexes with tridentate ligands. Cyclic TACN derivative—**L67** ligand was bound facially into the iron (III) complex, while 2,2′,6′,2″-terpyridine (**L68**) coordinated in a meridional mode. The X-ray structural analysis confirmed the molecular architecture and showed that due to its flexibility, **L68** could bind to metal in two coordination modes. Activity studies were carried out in dichloromethane with AgOTf in an oxygen atmosphere for 3 h. For all compounds, the conversion efficiency was almost 100%. For the **L67** complex, the conversion efficiency into extradiol products amounted to 97%, while for the **L68** complex, the ratio of 20% of intradiol to 78% of quinone products was determined. The obtained results showed that the coordination mode of tridentate ligands has implications for the cleavage mechanism [28].

Studies by Bruijnincx et al. showed that the iron (III) complex with the tripodal **L69** ligand is catalytically active. However, the reaction carried out in methanol or acetonitrile with air was extremely slow, and after about two weeks, only about 60% of the substrate was converted. The ratio (I/E) of intradiol and extradiol cleavage products was close to 1, which confirms that the composition of the first coordination sphere does not determine the reaction mechanism [67].

The fact that catechol dioxygenase has a histidine-rich metal environment inspired Wagner et al. to design a ligand **L70** structurally similar to **L69**. Complexes of **L70** were obtained and their molecular structure was confirmed with crystal structures. For one complex, the authors investigated the influence of solvent, the reaction time, and the addition of NaBPh_4_ on the catalytic activity and reaction efficiency. Results indicated that about 6 h is enough for 77–85% substrate conversion in most of the applied settings. Irrespective of the conditions tested, the amount of intradiol product (Figure 6) A was about 20–30%, whereas the ratio between extradiol products E, D, and product H changed substantially [68].

Among the tridentate ligands, compounds containing a boron heteroatom have been described by several groups. Structures of iron (III) complexes of ligands **L71** and **L72** were determined by X-ray structural analysis. The pyrazole rings in the ligands are substituted differently—**L71** has a *tert*-butyl and isopropyl substituents, while **L72** has two isopropyl groups. Despite having a five-coordinate sphere, the complex with **L71** turned out to be inactive towards dioxygen. In contrast, the six-coordinated complex with **L72** showed a catalytic activity via the extradiol cleavage mechanism [69]. This complex demonstrated an 85% catecholate conversion capacity. The analysis of conversion products revealed an almost two-fold excess of extradiol products over intradiol products. The obtained results proved that the steric effects of the substituents in the ligands are essential for determining the reaction mechanism for this type of complex [69]. Another five-coordinated iron (III) complex with **L74** was characterized by Yoon et al. [70].

Dhanalakshmi et al. examined the activity of complexes with meridionally bound ligands **L74**, **L75**, and **L68**. For the complex with **L74**, the main product is derivative B (57% of all products, Figure 6), while for the complex with **L75**, only 9.7% of different intradiol products were observed. For the **L68** complex, quinone **L** corresponded to 78% of all products (Figure 6) [71]. The obtained results are consistent with those by Jo et al. [28], who reported that *mer* complexes of tridentate ligands acted according to the intradiol or other (but not extradiol) mechanism [71]. Previous reports showed that *fac* isomers of iron complexes with the tridentate ligands are capable of converting catecholate according to the extradiol cleavage mechanisms. The iron (III) catecholate complexes with ligands **L76**, **L77**, **L78**, and **L79** were exposed to dioxygen in methanol and gave 80–90% substrate conversion. Only for the complex with **L79**, the amount of extradiol products was higher than intradiol products (E/I = 1.9). Among the tested ligands, only **L79** and a strong electron-withdrawing nitro group caused the electronic effects in the ring. The reduction of the electron density around the iron (III) facilitates the molecular attack of oxygen and hence the reaction according to the extradiol mechanism. These results confirm the significant influence of the nature of the Lewis base and the method of coordination [72].

Another series of tridentate **N_3_** compounds containing pyridine rings were investigated [24]. Among them, **L80** and **L81** formed *mer* isomers, whereas **L82** and **L83** *fac* isomers. For these chloride complexes, the influence of the solvent on the cleavage mechanism was tested. The intradiol cleavage mechanism prevailed for iron complexes with **L80**, **L83**, and **L81** in CH_2_Cl_2_, H_2_O, as well as micellar SDS (sodium dodecyl sulfate) and CTAB (cetrimonium bromide). For **L80** and **L83**, the largest amount of intradiol products was obtained in CH_2_Cl_2_ and SDS, and for **L81** in an aqueous solution (Figure 6B). Only for the iron (III) complex with L82, more extradiol products (Figure 6D,E) were formed, particularly in SDS. For facially bound ligands, products resulting from the other mechanism were also found. These results prove that the conversion efficiency and the course of the reaction depend on the reaction environment, Lewis acidity of iron (III), and the size and steric hindrance of the substituents in the ligand used.

Panda et al. described a series of **N_2_O** tridentate ligands, mimicking the architecture of the catechol dioxygenase active center. In the complexes, ligands **L84**–**L86** were bound to iron (III) in facial coordination mode. These complexes revealed high selectivity exceeding 80% of intradiol products and only 3% of extradiol products. DFT calculations performed for the established systems suggested that cleavage occurs with the formation of the iron (III) peroxide intermediate, which is consistent with the reaction mechanism for intradiol dioxygenases [73].

Váradi et al. published studies for six iron (III) complexes with coordinated isoindoline derivatives **L87**–**L92** (Figure 12). All **N_3_** donor ligands are bound to the coordination center in a meridional manner. As expected, the main products for all tested biomimetics were intradiol products (70%). The presence of larger substituents in isoindoline-based ligands is associated with increased reaction selectivity towards the intradiol mechanism. The flexibility of ligands and steric effects may be responsible for the extradiol product formation [74].

The series of linear **N_3_** donor ligands **L93**–**L97** with heterocyclic and aliphatic amine nitrogen donors showed a high selectivity of these 1,2-CD mimics. These ligands are facially bound in the complexes due to their size and electrochemical properties, creating favorable conditions for catalyzing the catechol conversion. The process efficiency was about 85–95%, and 88–90% of intradiol products were formed. The major cleavage product of catecholate was the derivative B (Figure 6) [75].

Interesting results were obtained for the iron (III) complex of **L93**, where a change of the solvent nature (e.g., use of micellar solvents) can switch the dominating conversion mechanism into the extradiol one. Using micellar SDS as a solvent, 93.7% of the extradiol products D, E (Figure 6) were obtained compared to 48.8% in water. As a result of the interaction of the cationic complex catalyst with an anionic solvent, it is possible to substitute chloride ligands with water molecules. This creates conditions required for binding molecular oxygen and mimicking the catalytic center of enzymes [25].

The catalytic properties of iron (III) complexes with facially bound ligands **L98** and **L99** (**L95** derivatives) and complexes with ligands **L100**–**L102** (**L96** derivatives) were investigated. The catalytic activity tests have shown that all complexes convert the substrate according to intradiol and extradiol mechanisms. In these complexes containing tridentate ligand and catecholate, the last place in the coordination sphere can be occupied by a chloride anion or a solvent molecule [76]. Other studies showed that iron (III) complexes with **L95** and **L96** ligands act according to the intradiol mechanism, and the chloride anion was the additional ligand in the coordination sphere [75]. For the rest of the complexes, the presence of chloride in the coordination sphere also enhances the intradiol mechanism, producing only several percent of extradiol products. For complexes containing a solvent molecule in the coordination sphere, the selectivity shifts towards the extradiol mechanism, with the highest yield of extradiol products detected for dichloromethane (E/I ratio from 7.2 to 18.5). The ratio of extradiol to intradiol products less than 1 (E/I <1) was found only for the processes carried out in DMF. The reaction selectivity was also increased by the replacement of pyridylmethyl arms with CH_2_NHR groups but decreased by the introduction of the hindering of N-alkyl substituents at the middle nitrogen atom in the ligand [76].

Sundaravel et al. investigated another group of ligands **L103**-**L106** bound facially. Iron (III) complexes converted catecholate according to intradiol and extradiol mechanisms. A high amount of extradiol products was formed for all compounds, with at least a two-fold excess over the intradiol products. The highest selectivity towards extradiol products was demonstrated for the iron (III) complex with **L104** (71%). However, the comparison showed that the complexes with tripodal **L62**–**L65** ligands gave the reaction rates more than 10 times higher. The presence of substituents in the ligand rings, their nature, and steric effects significantly affect the acidity of iron (III), which influenced the selectivity of the cleavage reaction [64].

Paria et al. reported the characterization and catalytic activity studies for the iron (III) complex with tridentate **N_2_O** ligand **L107**, a derivative of L-proline. The analysis of products of the 3,5-DTBC conversion showed a high reaction rate of about 80–90% for both dichloromethane and acetonitrile as solvents. The solvent change affects only the amount and not the ratio of extradiol to intradiol products (E/I), being close to 1. Notably, acetonitrile effectively prevents substrate conversion by other mechanisms, for example, auto-oxidation [77].

Visvaganesan et al. studied a series of iron (III) complexes with N-alkyl-substituted bis(pyrid-2-ylmethyl) amine ligands **L108**–**L114**. The crystal structure of complexes with **L109** and **L110** showed that the linear ligands facially bind to the metal center. The complexes showed the catalytic activity toward 3,5-DTBC in an oxygen atmosphere with AgClO_4_ as a chloride binding agent. Only for the complex with **L109**, the ratio of extradiol to intradiol products (E/I) was less than 1, and the intradiol product B (Figure 6) was the main product. For the remaining compounds, more extradiol products were obtained. Product E (Figure 6) turned out to be the main product of the reaction, and its highest contents were reported for the iron (III) complex with **L112** (46.1%). The highest E/I ratios (4.1–6.1) were obtained for complexes of ligands substituted with groups causing significant steric hindrances. The above results confirm that changing the acidity of iron (III) or introducing hindered groups is an efficient way to change the product selectivity [78].

Dhanalakshmi et al. investigated novel functionally active biomimetic iron (III) complexes with ligands derived from imidazole **L115**–**L117** and pyrazole **L118**–**L120**. The authors confirmed the dependence between the product selectivity and composition of the coordination sphere in the complex. For iron (III) complexes with **L115**–**L117**, changing the chloride anion into a solvent molecule (DMF) increases the selectivity towards the extradiol mechanism at least twice. On the other hand, ligands **L118**, **L120** bound in iron complexes did not show the ability to convert 3,5-DTBC according to intradiol and extradiol mechanisms, yet quinone products were formed [79].

Pyrazole derivatives, **L121**–**L124** ligands, were facially bound to iron (III), forming complexes that showed the ability to convert catecholate. Changing the medium from DMF to non-coordinating dichloromethane increased selectivity and efficiency towards the intradiol cleavage mechanism. The introduction of a sterically hindered group into the pyrazole rings increased the yield of extradiol products. Changing the methyl substituents to isopropyl increases the reaction rate, and the replacement of the pyrazole ring with an imidazole lowered the efficiency of the process [80].

The effect of the micellar medium was investigated for the iron (III) complexes with **L108**, **L68**, **L125**, **L126**. Complexes with **L108** and **L68** have been previously studied and showed an enhanced selectivity towards extradiol products [28,78]. Surprisingly, for the complex with ligand **L68**, no extradiol products were obtained in any tested media, whereas the main products were intradiol products. For the complex with coordinated **L108**, the latest reports are consistent with the previous studies where the observed selectivity towards the extradiol mechanism increased with introducing a micellar medium (SDS). The complex with the **L125** ligand showed no significant product selectivity, and the percentage content of various products was similar. However, for the complex with cyclic **L126**, the extradiol mechanism was favored, with the E/I ratio of at least 2 [26].

Iron (III) complexes with tridentate ligands constitute a larger group than tetradentate ligands, with the most abundant N_3_ donor ligands. Research indicates that the conversion mechanism of these complexes is influenced by many factors, the most important being *fac*/*mer* isomerism, electronic effects in the ligand affecting the Lewis acidity of central Fe ion, steric hindrance within the coordination sphere, and the solvent polarity. They seem to provide a valuable tool for the researcher because of the possibilities matching the process conditions to the expected products.

#### 3.1.3. Pentadentate Ligand

**Figure 13:**
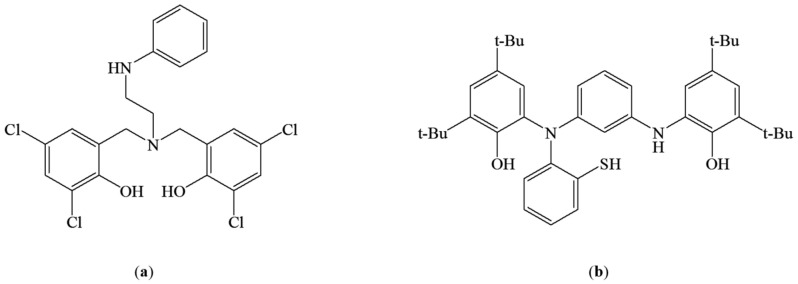
L127 (**a**) and L128 (**b**).

Metzinger et al. reported the unique iron (III) complexes with pentadentate N_2_O_2_S ligand **L127** (Figure 13a). The authors obtained two types of active complexes using different reaction conditions. The “normal” Fe complex was the result of synthesis with the use of KH and FeCl_3_. While Fe[N(SiMe_3_)_2_] was used as a substrate, the C–S bond was cleaved with the thiolate formation, which gave the rearranged ligand **L128** in the Fe complex (Figure 13b) [81]. The X-ray crystal analysis confirmed the architecture of both complexes. These complexes showed the ability to convert catecholate.

For the complex with **L127**, many more different products were isolated than for the complex with **L128**. Analysis of the catalytic activity of the **L127** complex revealed that using THF and dichloromethane as solvents, the 100% conversion was achieved after 24 h and 14 h, respectively. The main product in both reaction conditions was extradiol product D (27% and 59% for THF and DCM, respectively, Figure 6). For the **L128** complex, fewer different products were reported, and the activity tests were carried out in dichloromethane. The main product (55%) was again D (Figure 6), and the complete conversion was achieved after 14 h. However, for both complexes, products resulting from the intradiol and other mechanisms were identified [81].

#### 3.1.4. Influence of Solvent Properties on the Regioselectivity of Catechol Conversion

A search in the literature revealed that in few cases, the role of the solvent was also investigated. Due to the lack of detailed data in the comparable test systems, the definite comparison seems complicated. Nevertheless, some conclusions might be derived. Solvents forming stable complexes with the central Fe ion, such as DMF, could not be easily replaced by the substrate or dioxygen [24,26,57]. Therefore, such solvents would diminish the catalytic activity of the complex. In turn, removing labile-bound water from the Fe coordination sphere or using additional reagents to remove the Cl ligands would create a vacant position in the coordination sphere. Consequently, the ligand (three- or four-dentate) nature is orchestrated with the number of the possible vacant sites and determines the preferred intradiol or extradiol mechanism of the biomimetic action.

## 4. Conclusions

The 1,2-CD and 2,3-CD naturally occurring in bacteria were the inspiration for developing the biomimetic Fe complexes with similar catalytic activity. Although the catalytic mechanism for both enzymes was postulated, the precise mechanism of reactions catalyzed by the biomimetic complexes is not fully understood. Reports summarized in this review exhibited that the electronic effects of the substituents in the ligand affect the Lewis acidity of the metal center. The increasing Fe acidity enhances the ability to activate dioxygen and increases the catalytic activity towards the catechol conversion. In addition, studies indicate that conversion accelerators include the use of a deprotonating base and silver salts, removing a Cl^−^ anion from the coordination sphere. The ligand architecture and the substituent steric effects significantly affect the ability to bind the substrate in a monodentate and bidentate manner. As revealed by the published results, the substrate binding mode affects the conversion rate and determines the preferred mechanism and, consequently, the main products of the conversion. Initially, tetradentate ligands mimicking the active center of the enzymes were developed and tested. The presented analysis revealed that most of the tetradentate ligands show high intradiol selectivity. The vast majority of ligands have their tripodal architecture based on aromatic rings containing nitrogen atoms (pyridine and imidazole), which influence the Lewis acidity of the central atom. For mixed N/O ligands mimicking the catechol active site, the intradiol mechanism dominates. The extradiol mechanism was observed for tripodal ligands with steric hindrance caused by the pendant, for example, the aromatic moiety. The solvent polarity also affects the regioselectivity of the catalyst. For macrocyclic N_4_ ligands, the detected extradiol mechanism seems to result from the steric effects of the ligands, affecting the coordinating mode. Later tridentate ligands were proven to be active. For their complexes, the *fac* isomers favored the extradiol cleavage. For *mer* isomers, the primary mechanism was intradiol, but these complexes can oxidize the substrate as well, according to other mechanisms, e.g., auto-oxidation. Current results show the possibility of enhancing the specific catalytic mechanism with different solvent properties, in particular polarity. The anionic micellar medium (e.g., SDS - sodium dodecyl sulfate) strongly interacts with the positively charged catalysts, enhancing the interactions with the substrate and dioxygen. In turn, the cationic micellar medium CTAB (cetrimonium bromide) had a much weaker effect. The use of non-coordinating solvents, such as CH_2_Cl_2_, water coordination to the Fe center, or removal of Cl ligands from the coordination sphere, form the vacancy in the Fe coordination sphere and enhance the formation of the extradiol products. The literature reports also show that the strongly coordinating ligand (e.g., DMF - dimethylformamide) cannot be replaced by dioxygen, and in this way, the catalyst efficiency is decreased. The catechol dioxygenase architecture inspired chemists to synthesize biomimetics, which finally evolves to the broad spectrum of N/O donor ligands, revealing excellent catalytic activity.

This literature review shows the evolution of knowledge not only about CDs biomimetics. We can see new possibilities for controlling the regioselectivity and efficiency of the process by changing conversion conditions. With the structural analysis, we gained a powerful basis for designing efficient, durable, and universal catalysts, adjusting the mechanism depending on the user′s needs. Controlling the mechanism and the products obtained will improve the processes of removing pollutants with the simultaneous use of the compounds obtained. In addition, research indicates the possibility for used biomimetic iron (III) catalysts in many different processes.

## Data Availability

The data presented in this study are available on reasonable request from the corresponding author.
